# New Insights in Histogenetic Pathways of Gastric Cancer

**DOI:** 10.1097/MD.0000000000001810

**Published:** 2015-10-23

**Authors:** Simona Gurzu, Haruhiko Sugimura, Janina Orlowska, Zoltan Szentirmay, Ioan Jung

**Affiliations:** From the Department of Pathology, University of Medicine and Pharmacy of Tirgu-Mures, Romania (SG, IJ); Department of Tumor Pathology, Hamamatsu University School of Medicine, Hamamatsu, Japan (HS); Department of Pathology, Maria Sklodowska-Curie Memorial Cancer Centre and Institute of Oncology, Warsaw, Poland (JO); and Department of Molecular Pathology, National Institute of Oncology, Budapest, Hungary (ZS).

## Abstract

The aim of this paper was to describe 3 possible histogenetic pathways for poorly cohesive (diffuse) carcinomas and 2 for intestinal-type gastric carcinomas (GCs), which might influence the behavior of GC. In the present observational study, 102 patients with early (n = 50) and advanced GCs (n = 52) were evaluated, and the histogenetic background was analyzed. All of the cases were sporadic GCs. For particular aspects, Maspin, E-cadherin, and SLUG immunostains were performed. For our final conclusions, the results were correlated with literature data. In early stages, poorly cohesive carcinomas can display 3 histogenetic pathways, with particular molecular behaviors: “carcinoma with intraepithelial pagetoid onset” (with or without a switch from E-cadherin to SLUG positivity), “carcinoma with early lymphatic invasion” (carcinoma limited to mucosa but with carcinomatosis of the lymph vessels from subjacent layers), and “microglandular-type poorly cohesive carcinoma” (the onset is similar with adenocarcinoma but abrupt dedifferentiation can be seen in the submucosa, with persistence of a dual component in the deep layers). The intestinal type carcinoma can be developed on the background of superficially located dysplasia (“classic adenocarcinoma”) or in the submucosal heterotopic mucosa (“adenocarcinoma arising from the mucosal infolding in the submucosa”). Based on personal observations correlated with literature data, 5 histopathogenetic pathways are proposed with specific denominations. Each of them can partially explain the aberrant behavior of early gastric cancer.

## INTRODUCTION

Gastric carcinoma (GC) is a heterogenous tumor, histogenesis of which is not yet elucidated. Although the roles of *Helicobacter pylori,* intestinal metaplasia, and stepwise glandular dysplasia are agreed by most of the authors, the status of the background of gastritis remains unclear.^1,2^

To better understand the histogenesis, the histological aspect of early cancers and surrounding gastric mucosa should be attentively examined. Currently, the tumors limited to the mucosa (pT1a) or submucosa (pT1b), independent from the presence of lymph node metastases, are considered early gastric cancers (EGCs).^3–5^ Due to the increase of the metastatic risk based on the depth of infiltration, the superficial lesions are also grouped by the Japanese and European researchers in 6 distinct types: pT1a-m1 (invasion of the upper third mucosa), pT1a-m2 (middle third mucosa), pT1a-m3 (lower third mucosa), pT1b-sm1 (upper third submucosa), pT1b-sm2 (middle third submucosa), and pT1b-sm3 (lower third submucosa).^3,6,7^ Another classification that should be taken into account was performed by Kodama et al stating that based on the tumor growth, 3 groups of EGC were identified: small mucosal, superficially spreading (Super), and penetrating growth-type (Pen), with expansive (Pen A-type) or infiltrative growth (Pen B-type).^3,8^

However, the median time of progression from early to advanced stages of GC is ∼3.7 years and the steps of tumorigenesis are not well elucidated.^3^ Our observations, presented in this paper and correlated with previously known data, showed that at least 5 histogenetic pathways of GC can be identified and are distinct for poorly cohesive carcinomas and adenocarcinomas.

For a proper evaluation, we have used the adhesion marker E-cadherin, the indicator of epithelial-to-mesenchymal transition SLUG,^9^ the predictive marker HER-2, and the serine protease Maspin. Our previous results showed that Maspin protein is usually negative or present cytoplasmic positivity in the gastric mucosa; the cytoplasmic pattern is being also kept by the cells with intestinal metaplasia. The cytoplasmic positivity can be kept or a cytoplasmic-to-nuclear shift is seen in the dysplastic and tumor cells.^10^

## MATERIALS AND METHODS

The GC specimens included in this study were of patients from Romania, Japan, Hungary, and Poland. All the GC cases from Hamamatsu University School of University, Japan, were endoscopically-resected, whereas those from the Clinical County Hospital, Tirgu-Mures, Romania, National Institute of Oncology, Budapest, Hungary, and the Maria Sklodowska-Curie Memorial Cancer Centre and Institute of Oncology, Warsaw, Poland, were surgically removed tumors. Evaluation of the cases and publication of the results were approved by the heads of the fourth Clinics and the Ethical Committee of University of Medicine and Pharmacy of Tirgu-Mures, Romania.

To perform an observational study related on the histogenetic pathways of GC, the histopathological particularities of the 230 consecutive specimens from patients with GC who underwent surgical or endoscopic excision during 2003 to 2015 were reevaluated. For a proper examination, tumor tissue and normal peritumoral mucosa were necessary to be analyzed. To accomplish this objective, 102 form the 230 cases, in which the aspect of the peritumoral mucosa was possible to be analyzed on the same slide with the tumor tissue, were selected to be included in this study. None of the 102 patients presented with stump carcinoma or peptic ulcer, and none of them received preoperative oncotherapy. The cases crossing the serosa were also excluded. All of the 102 cases were sporadic GCs.

All the tissues were paraffin-embedded and were stained with Hematoxylin-Eosin, PAS-Alcian, SLUG (clone H-140, Santa Cruz Biotechnology - Heidelberg - Germany), E-cadherin (clone NCH-38, Dako - Glostrup - Denmark), HER-2 (clone 5A2 cerbB2-oncoprotein, Dako), and Maspin (clone EAW24, Novocastra - NewCastle upon Tyne - UK). For the immunohistochemical stains, the Novolink Polymer Detection System (Novocastra) was used. The unmasking antigen was used according to the instructions of the manufacturer and the developing was performed with DAB (diaminobenzidine) solution (Novocastra). For the negative controls, incubation was done with the omission of specific antibodies.^10^

The tumors were classified macroscopically according to the Japan Gastroenterological Endoscopy Society,^3–5^ whereas the depth of infiltration and microscopic tumor type were evaluated with the criteria proposed by the World Health Organization,^4^ American Joint Committee on Cancer,^5^ and Japanese guidelines.^3,6^

To identify the histogenetic pathways, the cases were first grouped in adenocarcinomas and poorly cohesive carcinomas that include signet-ring cell carcinomas (Table 1). Then, a careful examination of the tumor architecture and immunohistochemical particularities of the tumor cells and normal peritumoral mucosa, performed by all 5 experienced pathologists, was done. At the end, the observational aspects were discussed and correlated with literature data and a stepwise GC histogenesis was identified. In correlation with the data previously published in the literature by authors nominated in *References*, that also include our papers, we tried to establish specific denominations for each of the 5 possible histogenetic pathways of GC.

**TABLE 1:**
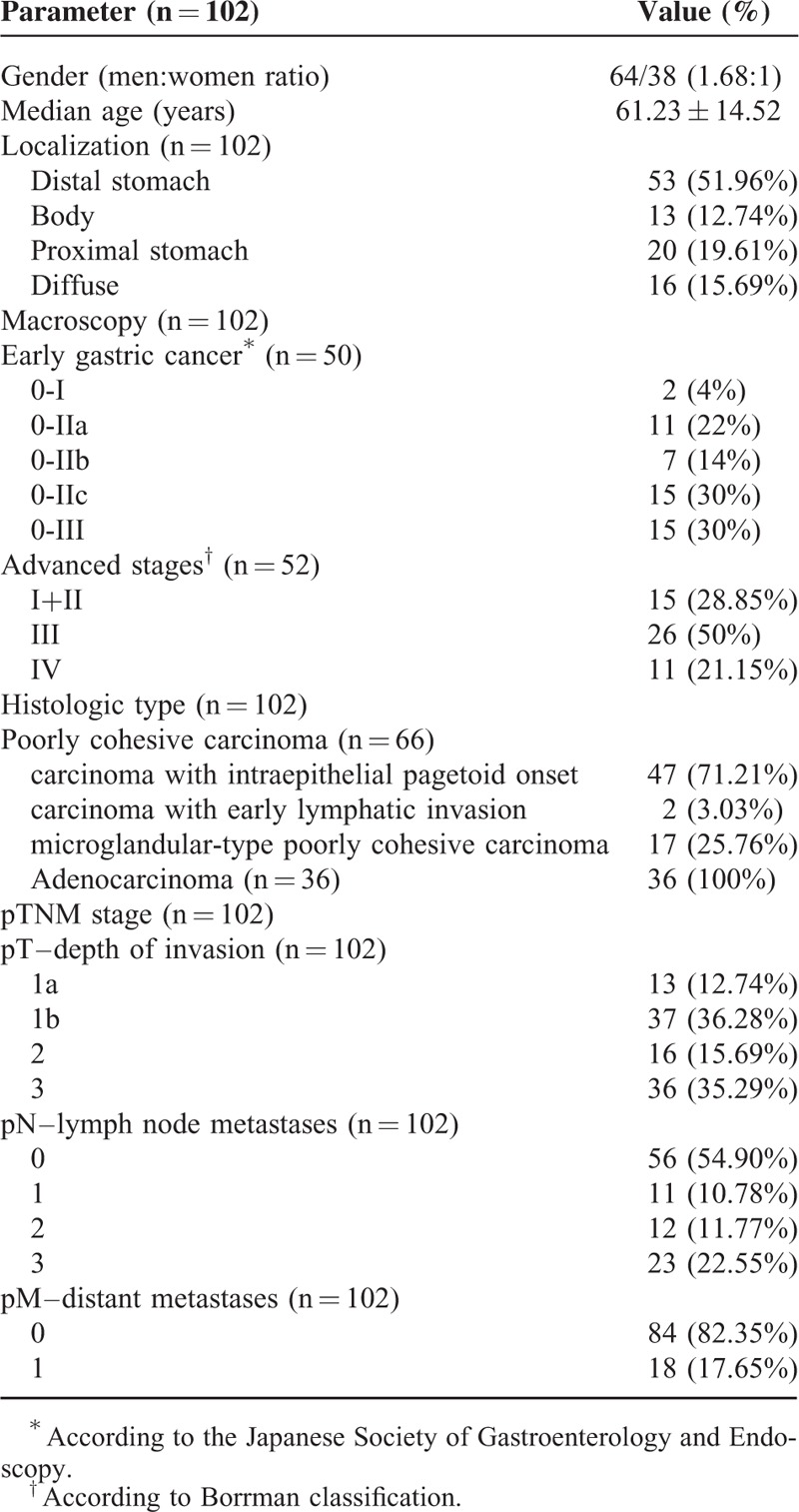
Clinicopathological Characteristics of the Patients

For this observational study, a descriptive statistic was performed using the GraphPad InStat3 program. The median age of the patients was presented adding the standard deviation value.

## RESULTS

### Clinicopathological Characteristics

From the 102 patients selected for this observational study, 50 were diagnosed with EGC, whereas the other 52 presented tumors with the invasion of the muscularis propria (pT2) or subserosal/serosal layers (pT3). The characteristics of the patients are summarized in Table 1.

### Histogenesis and Progression of Poorly Cohesive Carcinomas

In 47 from the 66 poorly cohesive carcinomas (Table 1), intramucosal pseudo-ring cell clusters were identified in the glandular layer, being covered by intact epithelium, independently from the tumor stage. These cases were considered to present a common histogenetic pathway and were nominated by our team as “carcinomas with intraepithelial pagetoid onset.” In two of the 66 cases an unusual carcinomatosis of the lymph vessels was observed and a second histogenetic pathway was described. Finally, a particular tumor spread was seen in 17 cases, showing a diffuse microglandular pattern. The particular aspects of histogenesis are presented below in this chapter.

### First Histogenetic Pathway—Carcinoma With Intraepithelial Pagetoid Onset

**FIGURE 1:**
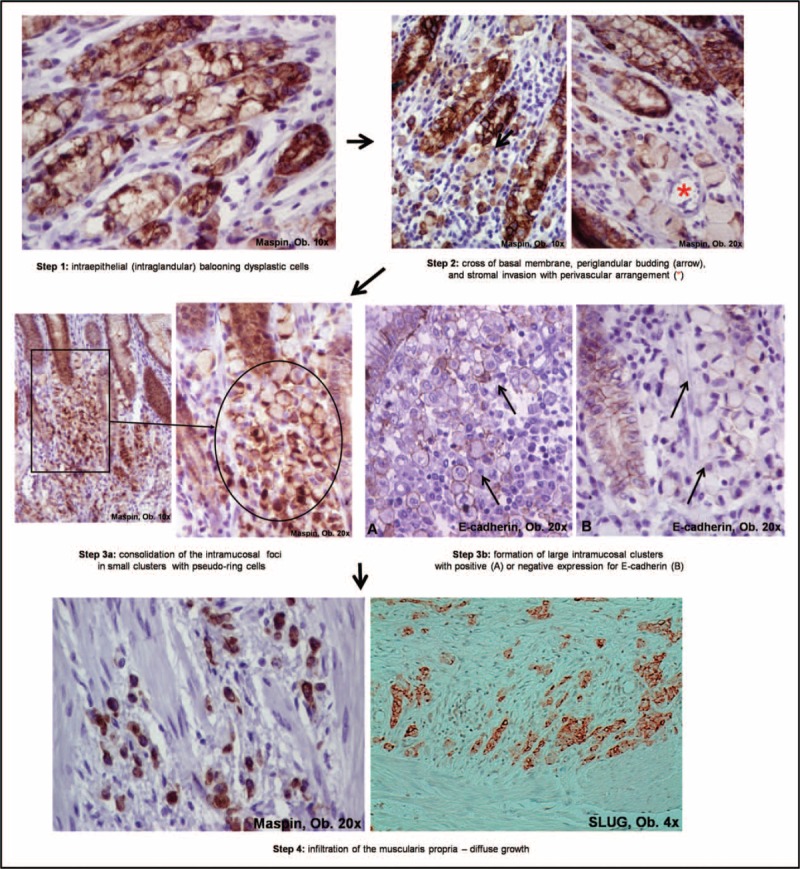
Carcinoma with intraepithelial pagetoid onset—the stepwise evolution.

The poorly cohesive carcinomas were mostly diagnosed in early stages, independently from the patient's age. The covering epithelium was intact and the tumor cells seemed to derive from the deep foveolar or glandular layer. The progression from early to advanced stages includes the following four steps (Fig. 1):

1. Step 1—intraepithelial (intraglandular) aggregation of tumor cells with clear cytoplasm and “ballooning aspect” that display pagetoid arrangement.
2. Step 2—crossing the basal epithelial membrane, with periglandular budding and invasion into the lamina propria. In this step, the tumor cells are isolated and can present radial arrangement around intramucosal blood vessels. They have clear cytoplasm and pseudo-ring cell aspect.
3. Step 3—intramucosal fusion of the isolated ballooning cells, with the formation of multicentric intramucosal foci. Further fusion of the small foci leads to the genesis of intramucosal clusters. The tumor cells inside the clusters can even keep E-cadherin positivity or lose its expression and gain SLUG positivity, as a sign of epithelial-mesenchymal transition.
4. Step 4—crossing of the muscularis mucosae and invasion of the subjacent layers. The tumor cells display a poorly cohesive architecture, with or without signet-ring cell component. They are usually HER-2 negative and present inconstant E-cadherin positivity.

### Second Histogenetic Pathway—Carcinoma With Early Lymphatic Invasion

**FIGURE 2:**
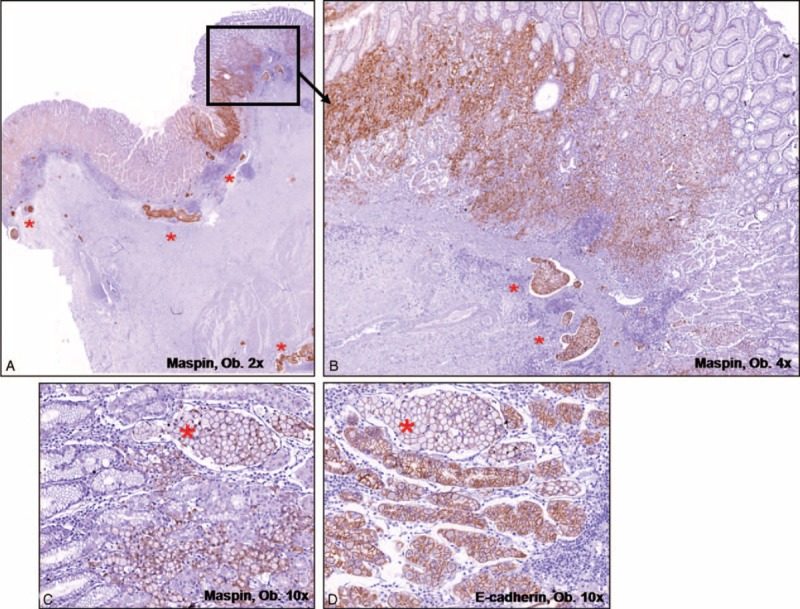
Carcinoma with early lymphatic invasion is limited to the mucosa but presents carcinomatosis of the lymph vessels (^∗^) in submucosa and muscularis propria (A, B). Inside the intramucosal lymph vessels (^∗^), Maspin (C), and E-cadherin expression is kept (D).

This unusual, extremely aggressive carcinoma is a variant of the “carcinoma with intraepithelial pagetoid onset.” The first 2 steps are similar to the first pathway, and tumor multicentricity is also a characteristic. The particularity of these carcinomas is that, during the 3rd step, the fusion of the clusters is more horizontal than vertical (Kodama's Super-type), and although they are limited to mucosa (pT1a), without the direct crossing of the muscularis mucosae, the early triggered invasion of the lymphatic channels of mucosa and submucosa is noted (carcinomatosis of the lymph vessels). The intramucosal and intravascular tumor cells keep E-cadherin and Maspin positivity and they are usually HER-2 negative. In 1 representative EGC diagnosed by our team, although the tumor was a pT1-a poorly cohesive carcinoma, the lymph vessels carcinomatosis was seen not only in the submucosa but also in the muscularis propria and serosa. Based on the current criteria the case was diagnosed as an EGC, but the lymph node and liver metastases were associated (Fig. 2).

### Third Histogenetic Pathway—Microglandular-Type Poorly Cohesive Carcinoma

**FIGURE 3:**
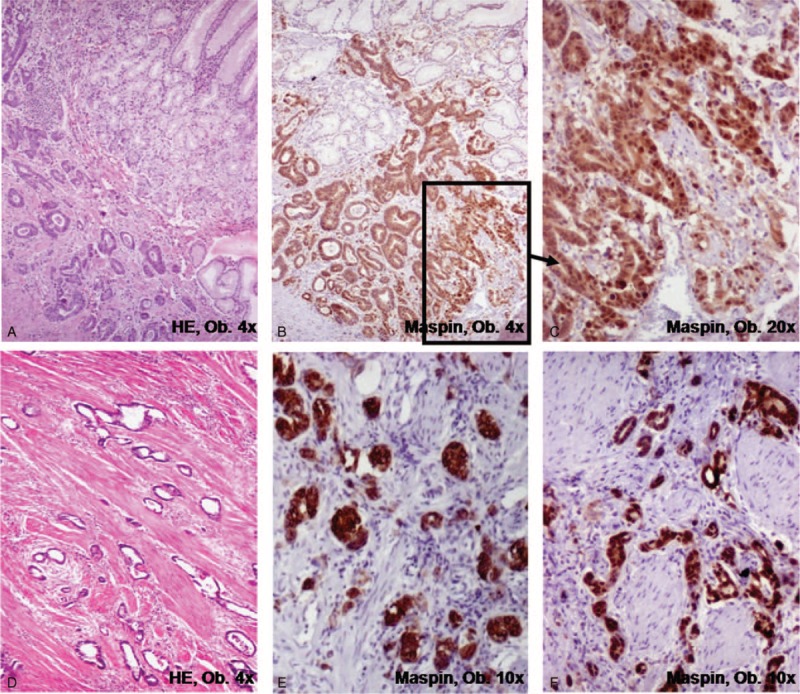
Microglandular-type poorly cohesive carcinoma presents intramucosal tubular architecture (A–C), with well-defined buddings (B,C), and diffuse growth with small glands in the muscularis propria (D–F).

Similar to the other 2 types of poorly cohesive carcinomas, this type also arise from the deep mucosal areas, being covered by intact epithelium. However, multicentricity is not a characteristic. Small glandular structures can be seen intramucosally, similarly to the onset of adenocarcinomas, but abrupt dedifferentiation with diffuse growth is noted after the invasion of the muscularis mucosae. In the submucosa and the deeper layers, the poorly cohesive architecture with proliferation of very small tubular structures (microglandular differentiation) is characteristic. The tumor cells that display a diffuse growth pattern are localized in a fibrotic stroma and display a pseudo-scirrhous aspect. These cells usually show a cytoplasmic Maspin and diffuse E-cadherin positivity in the superficial glandular component. Maspin nuclear switch, partial lost E-cadherin membrane expression, and SLUG positivity are seen in the cells infiltrating the deep layers (Fig. 3). HER-2 positivity can be inconstantly noted.

### Histogenesis and Progression of Adenocarcinomas

All the 36 adenocarcinomas included in the study (Table 1) shared a common classic pathway of development, in most of these cases the covering epithelium being ulcerated. However, possibility of genesis of adenocarcinomas from mucosal infolding in the submucosa should also be taken into account.

### First Histogenetic Pathway—Classic Tubular Carcinoma

**FIGURE 4:**
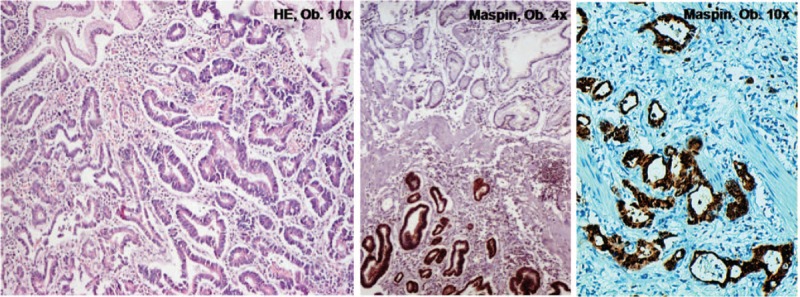
Classic tubular carcinoma presents a stepwise evolution from dysplasia to carcinoma, with progressive acquirement of Maspin positivity.

Most of adenocarcinomas are protruded or ulcerated tumors (types I, IIc, and III), and histogenesis involves both the foveolar and glandular layers. Dysplastic glandular structures with further malignant transformation are observed intramucosally in the first steps. Then, the formation of well-defined periglandular buds with Maspin cytoplasm to Maspin nuclear shift and inconstant E-cadherin/SLUG switch characterizes the invasion front (Fig. 4). A high density of the buds usually indicates strong local invasiveness, probably related to the epithelial-mesenchymal transition. HER-2 positivity is seen in >15% of the cases. Nodular growth is rarely encountered in the daily diagnosis, and the microglandular pattern does not occur. Also, the loss of Maspin expression is an indicator for an increasing risk of the distant metastases.^10^

### Second Histogenetic Pathway—Adenocarcinoma Arising From Mucosal Infolding in the Submucosa

**FIGURE 5:**
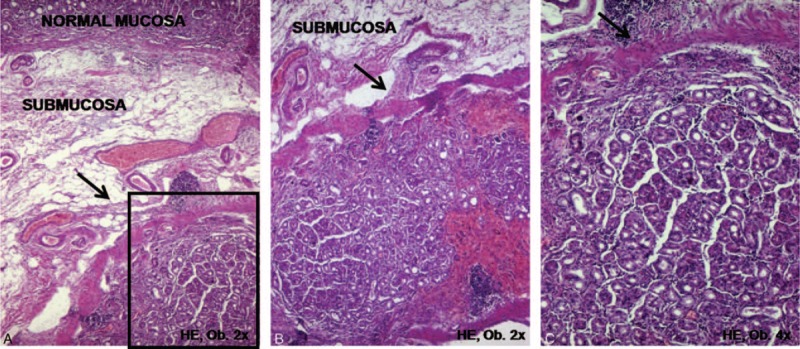
In the gastric submucosa, low power view (A,B) shows a round mucosal island surrounding by muscularis mucosae, with normal covering mucosa. A higher power view (C) shows no dysplastic or tumor cells but adenocarcinomas can originate from this mucosal infolding in the submucosal layer. In this case, the protruded lesion was endoscopically resected, in a 74-year-old men.

This type of carcinoma arises from the “heterotopic mucosa inside the submucosa” (Fig. 5), but its histogenesis is not clearly understood. We have few and ineloquent cases to properly characterize them. Some data from the literature are presented below.

## DISCUSSION

### Histogenesis and Progression of Gastric Adenocarcinomas

In line with the previous studies, classic adenocarcinomas seem to present a stepwise progression from chronic gastritis to dysplasia and adenocarcinoma.^1,2,11^ Intestinal metaplasia is observed in the peritumoral tissue in <50% of the cases and more frequent in elderly and patients with intestinal GC.^11^ There is gradual increase of the number of intestinal stem cells from normal to metaplastic and dysplastic cells in these cases.^12^

Unusual adenocarcinomas developing from “heterotopic gastric mucosa” located in the submucosa were first described by Iwanaga et al in 1975.^13^ The incidence of this heterotopy accounts for 3% to 20% in adults and is very rare in children and young people <20 years old.^13,14^ The mucosal infolding accompanied by the muscularis mucosae layer is more probably a result of *H. pylori*-related inflammation and not a congenital lesion.^13,14^ Based on this fact, it is rather considered to be an acquired lesion. The more appropriate term is “mucosal infolding in the submucosal layer” rather than “heterotopy.”^13,14^ Carcinomas with this origin are very rare considering, only a few cases being reported to date (0.02% of all GCs), most of them displaying a multicentric aspect.^14^ Malignant transformation of the infolded mucosa in the submucosa itself is extremely rare. Because it can lead to the protrusion of the covering mucosa, which is then more exposed to the inflammatory stimulus, it might be considered a para- rather than a premalignant lesion.^13,14^ Moreover, these heterotopic mucosa-related carcinomas growing deep in the submucosa are very rarely detected through endoscopy in early stages. An EGC developed from the submucosal infolded-mucosa which does not cross muscularis mucosae is histologically classified as an intramucosal (pT1a) carcinoma.^14^

### Histogenesis and Progression of Gastric Poorly Cohesive Carcinomas

The multicentric poorly cohesive carcinoma with intraepithelial pagetoid onset was mostly considered to be the characteristic for hereditary diffuse gastric cancer (HDGC) at the early onset^15–17^ or for *CDH1* germ-line mutation carriers.^18^ However, these *CDH1*-mutant tumors usually display signet-ring cell architecture and are E-cadherin negative.^15–18^ Being covered by an intact epithelial layer, these carcinomas are difficult to be detected through endoscopy in early stages.^10–15^ Based on the E-cadherin negativity, it was supposed that an initiation phase (Steps 1 and 2 in this study) is dependent on the destabilization of adherens junctions, whereas the progression phase (Steps 3 and 4) is based on the kinase-dependent induction of epithelial-mesenchymal transition.^18^ In our material that included sporadic tumors diagnosed in both the young and old, we did not notice any differences regarding the onset between E-caherin-positive and -negative cases. Moreover, although the intramucosal foci were considered to be formed by signet-ring cells,^10,15,16^ they seem to derive from the intraglandular “ballooning cells.” However, not all of them are marked by Alcian blue and their clustering below the submucosa can display nonsignet ring cell architecture. Therefore, we consider that their more appropriate denomination is “pseudo-ring cells.” The proliferation rate of these cells, quantified with Ki67, was proved to be lower than the rate in normal mucosa.^18,19^ The rapidly proliferating state might be related by other substances such as the Maspin protein.^10,18,19^

Although the intramucosal tumor foci were considered to have an indolent status,^18^ the aberrant behavior can be observed for type II of the poorly cohesive carcinomas, which were called “carcinoma with early lymphatic invasion” by our team. Their underlying genetic mechanism should be studied for further details.

The third type of poorly cohesive carcinomas was called “microglandular-type carcinoma” in this paper. Although it has an intramucosal onset similar to adenocarcinomas, an abrupt dedifferentiation with an aggressive behavior is seen in the early stages. Adenocarcinomas can also present dedifferentiation but it occurs in more advanced stages, in the deeper layers. Moreover, the “classic tubular carcinomas” are relatively indolent in their intramucosal stage.^18^

### Limitations of the Study and Final Remarks

The data presented in this paper showed the high complexity and heterogenic pathways of gastric cancer development. Understanding of different mechanisms could be of crucial importance to prognostic and therapeutic implications. The study limitations consist of the small number of cases that only allowed a descriptive statistic and observational correlations and did not allow examination of the geographic differences of the histogenetic pathways. However, its feasibility is based on the multicenter international collaboration and long-time experience in the field of GC of all the 5 pathologists included in the authors’ team. To prove these stepwise histogenetic pathways, experimental studies should be performed using specific gastric cancer cell lines.
